# *RNAlysis*: analyze your RNA sequencing data without writing a single line of code

**DOI:** 10.1186/s12915-023-01574-6

**Published:** 2023-04-07

**Authors:** Guy Teichman, Dror Cohen, Or Ganon, Netta Dunsky, Shachar Shani, Hila Gingold, Oded Rechavi

**Affiliations:** 1 Department of Neurobiology, Wise Faculty of Life Sciences and Sagol School of Neuroscience, Tel Aviv University, Tel Aviv, Israel; 2 Department of Biology, Technion – Israel Institute of Technology, Haifa, Israel; 3 Sagol Brain Institute, Sourasky Medical Center, Neurological Institute, Tel Aviv and Sagol School of Neuroscience, Tel Aviv University, Tel Aviv, Israel; 4 Sackler Faculty of Medicine, Tel Aviv University, Tel Aviv, Israel

**Keywords:** RNA sequencing, Clustering analysis, Gene set enrichment analysis, Data visualization, Pipeline, Graphical interface, Differential expression, Computational analysis

## Abstract

**Background:**

Among the major challenges in next-generation sequencing experiments are exploratory data analysis, interpreting trends, identifying potential targets/candidates, and visualizing the results clearly and intuitively. These hurdles are further heightened for researchers who are not experienced in writing computer code since most available analysis tools require programming skills. Even for proficient computational biologists, an efficient and replicable system is warranted to generate standardized results.

**Results:**

We have developed *RNAlysis*, a modular Python-based analysis software for RNA sequencing data. *RNAlysis* allows users to build customized analysis pipelines suiting their specific research questions, going all the way from raw FASTQ files (adapter trimming, alignment, and feature counting), through exploratory data analysis and data visualization, clustering analysis, and gene set enrichment analysis. *RNAlysis* provides a friendly graphical user interface, allowing researchers to analyze data without writing code. We demonstrate the use of *RNAlysis* by analyzing RNA sequencing data from different studies using *C.* *elegans* nematodes. We note that the software applies equally to data obtained from any organism with an existing reference genome.

**Conclusions:**

*RNAlysis* is suitable for investigating various biological questions, allowing researchers to more accurately and reproducibly run comprehensive bioinformatic analyses. It functions as a gateway into RNA sequencing analysis for less computer-savvy researchers, but can also help experienced bioinformaticians make their analyses more robust and efficient, as it offers diverse tools, scalability, automation, and standardization between analyses.

**Supplementary Information:**

The online version contains supplementary material available at 10.1186/s12915-023-01574-6.

## Background

RNA sequencing continues to grow in popularity as an investigative tool for biologists. A vast variety of RNA sequencing analysis methods allow researchers to compare gene expression levels between different biological specimens or experimental conditions, cluster genes based on their expression patterns, and characterize expression changes in genes involved in specific biological functions and pathways.

Specific tools exist to perform the tasks described above (see the Discussion section and Additional File 1: Table S1 for a detailed comparison of available tools). However, most analysis tools can only perform a subset of these tasks. Any out-of-the-ordinary research questions require researchers to write customized analysis scripts, which may not be easy to share or replicate. Moreover, many of the existing tools require users to be familiar with reading and writing code, making them usable only by researchers experienced in computer programming.

*RNAlysis* offers a solution to these problems by (1) using a modular approach, allowing users to either analyze their data step-by-step, or construct reproducible analysis pipelines from individual functions; and (2) providing an intuitive and flexible graphical user interface (GUI), allowing users to answer a wide variety of biological questions, whether they are general or highly specific, and explore their data interactively without writing a single line of code. *RNAlysis* includes thorough documentation and step-by-step guided analyses, to help new users to learn the software quickly and acquire good data analysis practices (available online or as Additional File 2: Tutorial).

## Implementation

**Fig. 1:**
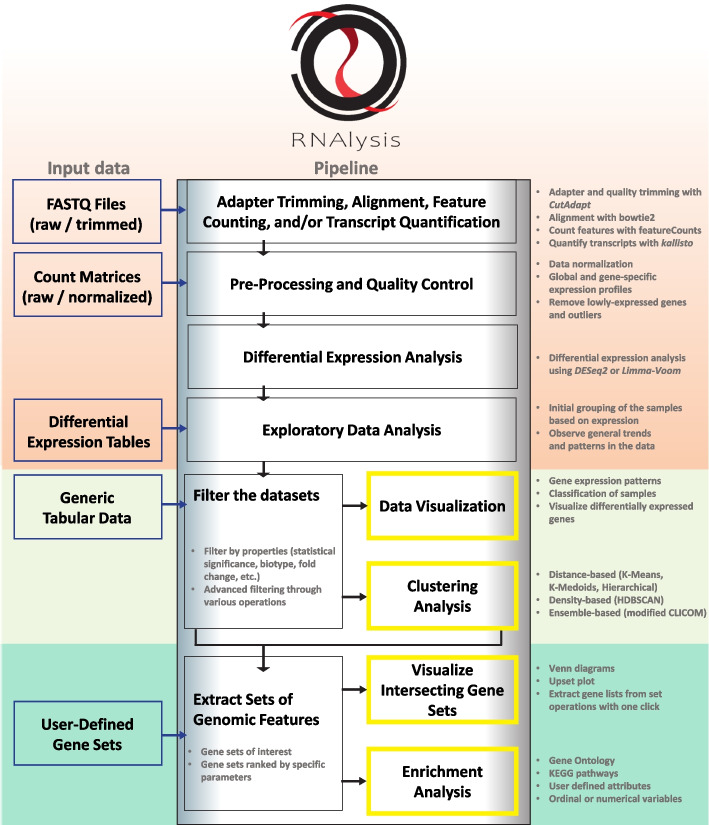
The workflow of *RNAlysis.* **Top section:** a typical analysis with *RNAlysis* can start at any stage from raw/trimmed FASTQ files, through more processed data tables such as count matrices, differential expression tables, or any form of tabular data. **Middle section:** data tables can be filtered, normalized, and transformed with a wide variety of functions, allowing users to clean up their data, fine-tune their analysis to their biological questions, or prepare the data for downstream analysis. *RNAlysis* also provides users with a broad assortment of customizable clustering methods to help recognize genes with similar expression patterns, and visualization methods to aid in data exploration. All of these functions can be arranged into customized Pipelines that can be applied to multiple tables in one click, or exported and shared with others. **Bottom section:** Once users have focused their data tables into gene sets of interest, or imported such gene sets from another source, they can use *RNAlysis* to visualize the intersections between different gene sets, extract lists of genes from any set operations applied to their gene sets and data tables, and perform enrichment analysis for their gene sets, using either public datasets such as GO and KEGG or customized, user-defined enrichment attributes

*RNAlysis* was designed to perform three major tasks: (1) pre-processing and exploratory data analysis; (2) finding gene sets of interest through filtering, clustering, and set operations; (3) visualizing intersections between gene sets and performing enrichment analysis on those sets (Fig. 1).

### Input

*RNAlysis* can interface with existing tools, such as *CutAdapt*, *kallisto*, *bowtie2*, *featureCounts*, *limma*, and *DESeq2* [1–8], to enable users to run basic adapter-trimming, RNA sequencing quantification, read alignment, feature counting, and differential expression analysis through a graphical user interface. That is to say, users can begin their analysis with *RNAlysis* with sequencing data at any stage. Alternatively, users can load into *RNAlysis* data tables that were generated elsewhere. *RNAlysis* has a tab interface, which allows users to examine and analyze multiple data tables in parallel, seamlessly switching between them.

*RNAlysis* can accept data from any organism. *RNAlysis* can analyze gene expression matrices (raw or normalized), differential expression tables, or user-defined gene sets of interest. Moreover, *RNAlysis* accepts annotations for user-defined attributes of genes. Since *RNAlysis* works with tabular data, *RNAlysis* is applicable to any type of data table.

### Data validation and pre-processing

First, *RNAlysis* allows users to validate their data by summarizing and visualizing its patterns and distribution. For instance, users may compare the distribution of gene expression between samples through scatter plots and pair plots, or examine general trends in the data, as well as potential batch effects, via clustergram plots and PCA projections.

Moreover, *RNAlysis* allows users to pre-process their data by normalizing it through one of the various methods (such as median of ratios, relative log ratio, trimmed mean of M-values, and more) [3, 9–12], filtering out lowly-expressed genes, and eliminating rows with missing data from their tables.

### Data filtering and clustering

After data pre-processing, users can filter their data tables according to a broad array of parameters, depending on the nature of their data and biological questions. These filtering functions can be applied in particular orders and combinations to suit the user’s specific needs. These functions include, among many others, filtering by statistical significance and/or the direction and magnitude of fold change, filtering genomic features by their type, performing set operations between different data tables and gene sets (for instance — intersections, differences, majority vote intersections, and so on) between tables, etc.

One of the powerful features of *RNAlysis* is the ability to easily extract gene lists from set operations applied to the user’s tables and gene sets, and use these lists in downstream analyses. Users can do this by applying a pre-defined set operation (like intersection or difference) or by hand-picking subsets of interest through an interactive graphical platform.

Finally, *RNAlysis* allows users to cluster genes based on the similarity of their expression patterns. *RNAlysis* supports an extensive selection of clustering algorithms, including distance-based clustering (K-Means, K-Medoids, Hierarchical clustering), density-based clustering (HDBSCAN) [13], and ensemble-based clustering (a modified version of the CLICOM algorithm) [14].

Moreover, *RNAlysis* provides users with a wide array of distance metrics for clustering analysis. This includes the implementation of distance metrics that were specially developed for biological applications, such as time-course gene expression data [15], and distance metrics that were empirically found to best suit transcriptomics analysis [16].

### Modularity and building customized pipelines

Filtered data tables can be saved or loaded at any stage during the analysis. The operations performed on the data and their order will be automatically reflected in the output files' names. Additionally, any operation applied to the data can be undone with a single click, and *RNAlysis* displays the history of commands applied to each table in the order they were applied.

As mentioned earlier, users can “bundle” any of the functions *RNAlysis* offers into distinct Pipelines, which can then be applied in the same order and with the same parameters to any number of similar data tables. This helps users to save time and avoid mistakes and inconsistencies when analyzing a large number of datasets. Pipelines can also be exported and shared with other researchers, who can then use these Pipelines on any machine that installed *RNAlysis*. This feature makes analysis pipelines easier to report and share, increasing the reproducibility and transparency of bioinformatic results.

### Enrichment analysis

After applying the analyses mentioned above to summarize data tables down to gene sets of interest, users can carry out enrichment analysis for those gene sets. Gene set enrichment analysis is a collection of methods for identifying classes of genes, biological processes, or pathways that are over- or under-represented in a gene set of interest [17]. Enrichment analysis is highly prevalent in RNA sequencing analysis since it allows researchers to associate a differentially-expressed gene set with underlying biological functions [18, 19].

*RNAlysis* supports multiple approaches and statistical methods for enrichment analysis, including classic gene set enrichment analysis, permutation tests [20], background-free enrichment analysis [21, 22], and enrichment for ordinal or continuous variables.

*RNAlysis* can automatically retrieve enrichment analysis annotations of all major model organisms from widely accepted databases such as Gene Ontology categories and KEGG pathways [23, 24]. However, unlike many other analysis pipelines, *RNAlysis* also accepts annotations for user-defined attributes and groups (see Additional File 1: Table S1). This allows users to tailor their analyses to their specific needs and biological questions.

### Documentation and accessibility

While *RNAlysis* can be operated entirely within a graphical interface, all the functions and features *RNAlysis* offers can also be imported and used in standard Python scripts, allowing users with coding experience to further automate and customize their bioinformatic analyses. Pipelines that are generated with the graphical interface can be imported and used through the programmatic interface and vice versa, maximizing the reproducibility and shareability of analyses performed with *RNAlysis*.

Moreover, whenever *RNAlysis* integrates with external tools and packages for tasks such as alignment or differential expression, *RNAlysis* will export the exact command line or R script used to produce the results, allowing all users to share the full details of their analysis.

*RNAlysis* includes extensive documentation to guide new and returning users. A user guide and a tutorial offer a bird’s eye view of the modules and features of *RNAlysis*, along with video demonstrations, usage examples, and recommended practices. A frequently asked questions page offers solutions to common questions and issues posed by users of *RNAlysis*. The remainder of the documentation provides a complete reference of the functions and features available in *RNAlysis*. Users can look up specific entries for a more thorough review of their theoretical background, use cases, and optional parameters.

*RNAlysis* can either be installed as a Python package on all standard operating systems, or downloaded as a stand-alone application, which does not require users to install any mandatory dependencies. This simplifies the acquisition process of *RNAlysis*, eliminating the initial barrier of entry for less computationally-oriented users.

The project is available as an open-source, public GitHub repository. Numerous test cases are also provided within the package, which are executed automatically every time the source code is updated, ensuring that data analysis with *RNAlysis* remains consistent and reliable.

*RNAlysis* is powered by various open-source projects [15, 25–34] which are installed automatically and used when needed.

## Results

We examined the ability of *RNAlysis* to facilitate analyses of multiple different publicly available datasets [35–38]. First, we analyzed time-series gene expression data, using clustering analyses to group genes based on their expression pattern. Then, we demonstrated the analysis of multiple RNA sequencing datasets from raw FASTQ files, showing the applications of analysis Pipelines and set operations between datasets. A step-by-step tutorial of these analyses is available in the online *RNAlysis* documentation (also available as Additional File 2: Tutorial).

### Analysis #1: Exploring gene expression patterns across the development of *Caenorhabditis elegans*

In the first analysis, we examined a dataset describing average gene expression under different developmental stages of *Caenorhabditis* *elegans* nematodes, derived from the control samples of many publicly available RNA sequencing experiments [35].

**Fig. 2:**
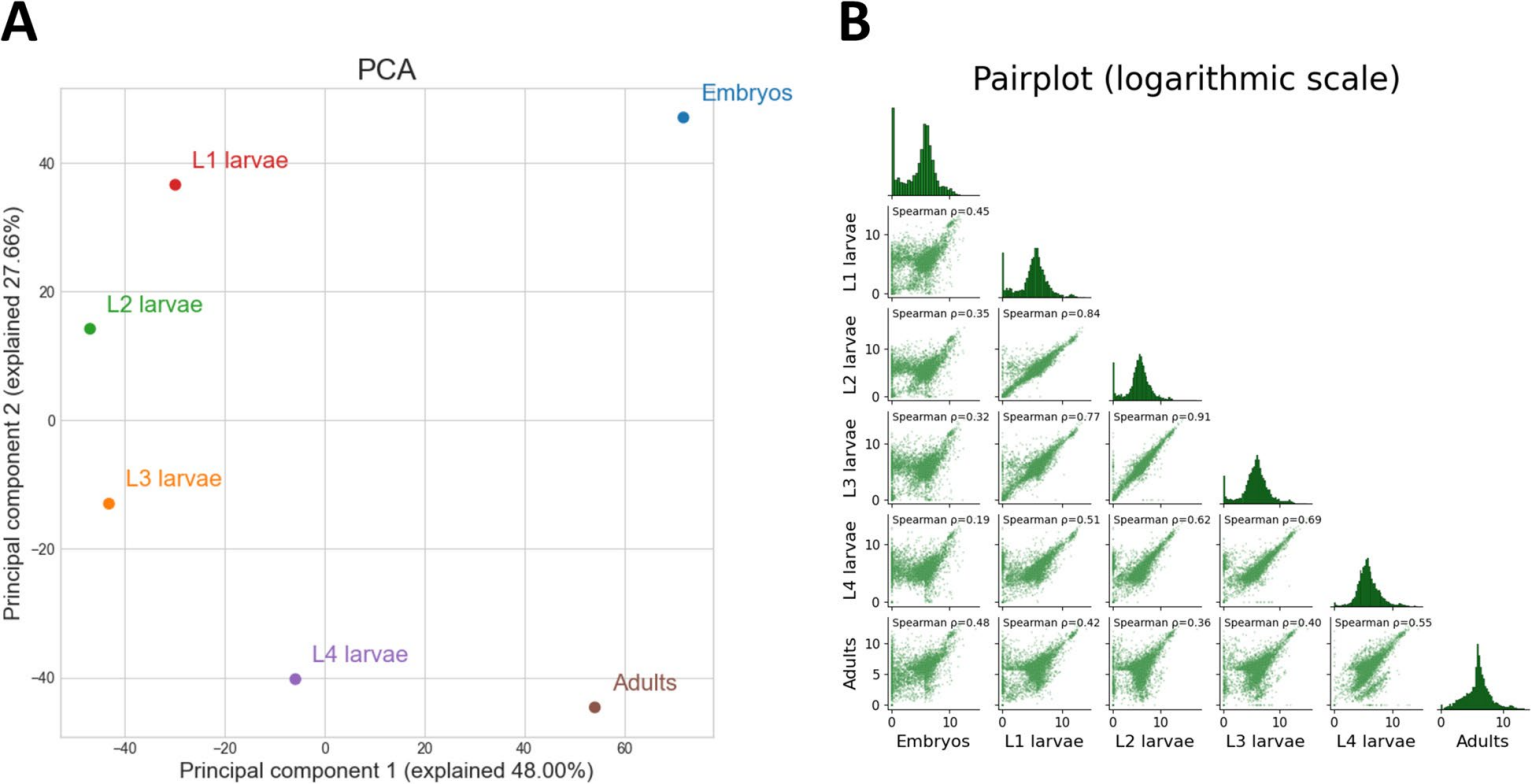
Exploratory data analysis reveals patterns in time-series gene expression data. **A** Principal component analysis projection of the time-series data. Depicted are the first two principal components, explaining > 75% of the variance in the data. Data was power-transformed and standardized before the analysis. **B** Pair-plot, depicting the pairwise Spearman correlation between each pair of samples, and a histogram of normalized gene expression in each sample. Each dot represents the log of normalized expression of a single gene

Exploratory data analysis revealed that the different developmental stages show the highest correlations between contiguous developmental stages (Fig. 2A), and PCA uncovered a semi-circular pattern, with over 75% of the data’s variance explained by the first two principal components (Fig. 2B). Interestingly, the first principal component arranges the samples by their relative germline content, with embryos and adult nematodes on one end, L1–L3 larvae on the other, and L4 larvae in between. The second principal component arranges the samples by their developmental stage, with embryos at the top of the graph and adults at the bottom.

**Fig. 3:**
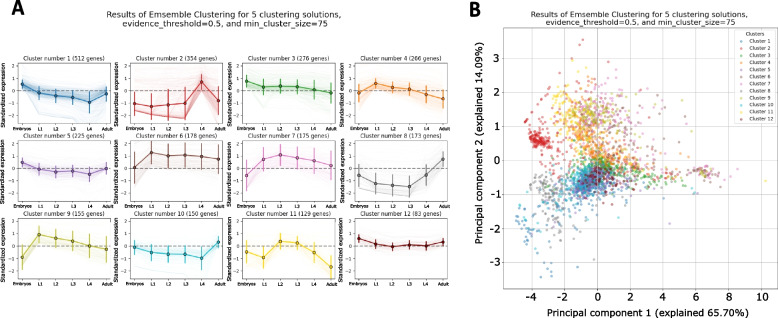
Clustering analysis of time-series gene expression data. **A** Clustering analysis of the data using modified CLICOM clustering, using five underlying clustering setups, an evidence threshold of 50%, and a minimal cluster size of 75 [14]. Clusters are sorted by their size. Each graph depicts the power-transformed and standardized expression of all genes in the cluster, with the center lines denoting the clusters’ means and standard deviations across developmental stages of *C.* *elegans* nematodes. **B** PCA projection of the power-transformed and standardized gene expression data. Each dot represents a gene. The points are colored according to the cluster they belong in the CLICOM clustering result depicted in **A**

Next, we extracted clustering results at three different resolutions by using exemplars from three different classes of clustering algorithms: a distance-based algorithm (K-Medoids) (Additional File 3: Figure S1), a density-based algorithm (HDBSCAN) (Additional File 4: Figure S2), and an ensemble-based algorithm (CLICOM) (Fig. 3). While one of the most challenging aspects of RNA sequencing clustering analysis is the requirement to specify in advance the number of clusters, *RNAlysis* provides unbiased clustering methods that can either estimate a good number of clusters to detect, or require no such input at all — instead specifying the smallest cluster size that would be of interest to the user.

While the data examined here only contains a single entry for each experimental condition, *RNAlysis* is well suited for clustering analysis of replicate data, since it's able to cluster each batch of replicates separately and combine the results of those batches, resulting in more accurate and robust clustering results [39].

**Fig. 4:**
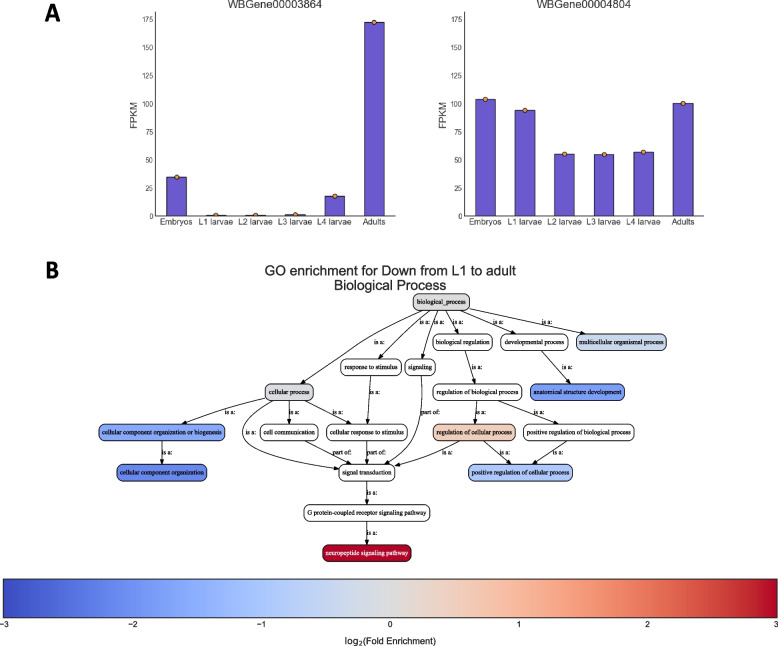
Gene expression plots and enrichment analysis of time-series gene expression data. **A** Normalized gene expression values of the time-series data for the two sample genes *oma-1* (WBGene00003664) and *skn-1* (WBGene00004804). **B** GO enrichment ontology graph, depicting enrichment results for cluster #9 (genes whose expression decreases along development from L1s to adults) (see Fig. 3). The graph depicts the hierarchical relationship between the GO terms. Each GO term was colored according to its log_2_ (Fold Enrichment) score if it was statistically significant (*q*-value ≤ 0.05)

Finally, we plotted the expression level of specific genes of interest under the different developmental stages (Fig. 4A) and performed GO enrichment analysis on one of the clusters we previously detected, revealing a robust enrichment for neuropeptide signaling pathways (Fig. 4B).

### Analysis #2: Measuring the effect of stress on the expression of small RNA factors

In the second analysis, we analyzed three datasets that examined the effects of three different stress conditions (osmotic stress, heat shock, and starvation) on gene expression [36–38]. This is a replication of our previously published analysis [40] done with an earlier version of *RNAlysis* (version 1.3.5, 2019), where the purpose was to examine the effects of stress exposure on the expression of small RNA factors. This analysis shows how *RNAlysis* facilitates answering highly specific biological questions in an intuitive manner.

We started the analysis with raw FASTQ files, applying adapter trimming, pseudo-alignment, transcript expression quantification, and differential expression analysis to the three datasets, all executed through the *RNAlysis* graphic interface.

**Fig. 5:**
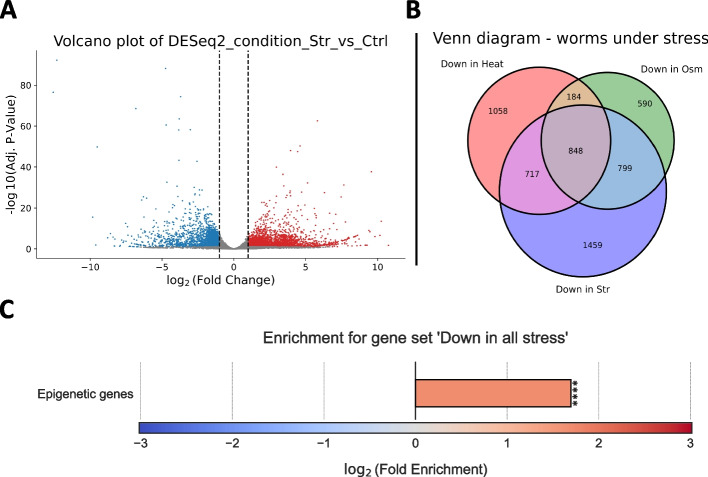
Analysis of stress-induced gene expression changes. **A** Volcano plot depicting differential expression results, comparing worms that experienced starvation (str) to worms that grew under normal conditions. Each dot represents a gene. Differentially expressed genes with log_2_ (fold change) ≥ 1 were painted in red, and differentially expressed genes with log_2_ (fold change) ≤  − 1 were painted in blue. **B** A proportional Venn Diagram depicting the intersections between genes that are significantly downregulated under heat shock, osmotic stress, or starvation, compared to their matching control samples. **C** Log_2_ (Fold enrichment) score for a curated list of epigenetic genes, in the set of genes significantly downregulated under all stress conditions. The *p*-value for enrichment was calculated using 10,000 random gene sets identical in size to the tested group. *** indicates adj. *p*-value ≤ 0.001

Next, we examined the distribution of differentially expressed genes under each condition with a Volcano Plot (Fig. 5A) and extracted from each differential expression table the lists of significantly up-regulated and down-regulated genes. This step was automated by building and applying a Pipeline, allowing us to analyze all three tables in the exact same manner with the click of a button.

Following these filtering steps, we examined the intersection of the up- and down-regulated genes between the different stress conditions (Fig. 5B) and extracted the list of genes that are significantly up/down-regulated under all stress conditions. We then created an appropriate background set for enrichment analysis by calculating the union gene lists of all genes which are sufficiently expressed under at least one stress condition.

Finally, we ran an enrichment analysis on the stress-downregulated genes, measuring whether they are significantly enriched for a user-defined list of epigenetic-related genes (Fig. 5C). We found the stress-downregulated genes to be significantly enriched for epigenetic-related genes, as previously shown [40]. Enrichment for user-defined attributes is a feature unique to *RNAlysis*, allowing users to answer highly specific biological questions. This means that the users are not limited to widely available datasets, but can directly analyze any gene sets and attributes of interest without the need to write any code.

## Discussion

*RNAlysis* offers researchers a robust, scalable, and easy-to-use tool to analyze RNA sequencing data. *RNAlysis* was designed not only to be intuitive and approachable for new users, but also to provide a high degree of efficiency, control, and robustness to experienced bioinformaticians.

Other useful software tools for the analysis of RNA sequencing data exist (see Additional File 1: Table S1). For example, *Galaxy* [41, 42] is a web-based scientific analysis platform for the analysis of biological data. *Galaxy* offers many shared features with *RNAlysis*, including integration of existing analysis tools, extensive documentation, shareable pipelines, and the ability to filter, sort, and intersect data tables. Galaxy has a large following [42] and supports a wide array of established tools from various fields of bioinformatics. Contrary to *Galaxy*, *RNAlysis* aims to simplify commonly used actions for RNA sequencing analysis in particular, such as filtering and set operations. This is done by providing users with dozens of ready-made filtering functions relevant to RNA sequencing data and supporting set operations on an arbitrary number of datasets with an intuitive, point-and-click interface. Moreover, *RNAlysis* offers analysis methods that are especially useful to RNA sequencing data, such as advanced clustering methods and enrichment analysis for user-defined attributes, which are not available on *Galaxy*.

Tools such as *ARPIR* [43] and *NetSeekR* [44] can take users all the way from the alignment of reads and differential expression analysis through GO enrichment and other tertiary analyses such as gene network analysis. Other tools like *ideal* [45], *PIVOT* [46], and *DEBrowser* [47] provide users with a graphical interface to perform differential expression analysis and enrichment analysis.

While these tools allow less experienced bioinformaticians to perform basic transcriptomic analysis, they are limited in their capability to filter datasets, perform set operations between datasets, use more advanced clustering algorithms, or automate and streamline data analysis with pipelines. In contrast to these tools, *RNAlysis* is highly modular and customizable, allowing users to tailor their investigations to their biological questions through advanced data filtering, intersecting multiple datasets, and a high degree of control over analysis parameters at every stage of the process. Moreover, *RNAlysis* can analyze RNA sequencing experiments from start to finish since it supports pre-processing, alignment, and quantification utilities of FASTQ files.

## Conclusions

*RNAlysis* offers a modular toolbox for RNA sequencing data analysis, with the unique combination of an intuitive graphical interface and highly customizable analysis workflows, making it distinct from many other RNA sequencing analysis tools.

We believe that the ability to build customized and reproducible analysis pipelines, combined with the user-friendly interface, will allow researchers to gain novel biological insights from RNA sequencing data easily.

## Methods

For a detailed description of the analysis pipelines used to generate the results displayed here, see Additional File 2: Tutorial.

### Statistical analysis

GO Enrichment analysis (Fig. 4) was performed as follows: GO annotations were retrieved through the GO Solr search engine API called GOlr. Annotations were filtered using the parameters described in Additional File 2: Tutorial: kept only annotations for *C.* *elegans* (taxon ID 6239), excluded “NOT” annotations. Annotations were propagated using the ELIM algorithm [48]. *P*-values were calculated using the hypergeometric test, and corrected for multiple comparisons using the Benjamini/Yekutieli FDR method for general or negatively correlated tests [49].

Enrichment for user-defined attributes (Fig. 5) was performed as follows: Annotations were generated as described in Additional File 2: Tutorial. P-value was calculated using a permutation test, using 10,000 random gene groups identical in size to that of the examined group of differentially expressed genes. *P*-value was then calculated using the formula *p* = *(successes* + *1)/(repeats* + *1)*, where success are defined as randomized gene set with a proportion of positively-annotated genes equal to or larger than the proportion in the test set. This formula results in a positively-biased estimator of the real *p*-value (a conservative estimate of the *p*-value).

## Availability and requirements

**Project name:** *RNAlysis.*

**Project home page:** https://github.com/GuyTeichman/RNAlysis

**Operating system(s):** Platform independent

**Programming language:** Python 3

**Other requirements:** Python 3.7.9 or higher (optional), GraphViz 3.0 or higher (optional), kallisto 0.44.0 or higher (optional), bowtie2 2.3.5 or higher (optional), R 4.0.0 or higher (optional), Microsoft C++ Build Tools 14.0 or higher (optional, on Windows computers only), Perl 5.9 or higher (optional)

**License:** MIT

Any restrictions to use by non-academics: none

## Supplementary Information

**Additional file 1: Table S1.** Comparison between RNAlysis and other existing analysis tools.

**Additional file 2:** Tutorial.

**Additional file 3: Figure S1.** K-Medoids Clustering analysis of time-series gene expression data. Clustering analysis of the data using K-Medoids clustering, after selecting an appropriate number of clusters (K = 11) using the Gap Statistic method [50]. Clusters are sorted by their size. Each graph depicts the power-transformed and standardized expression of all genes in the cluster, with the center lines denoting the clusters' Medoids and standard deviations across developmental stages of *C. elegans* nematodes.

**Additional file 4: Figure S2.** HDBSCAN Clustering analysis of time-series gene expression data. Clustering analysis of the data using HDBSCAN clustering, with a minimal cluster size of 75 [13]. Clusters are sorted by their size. Each graph depicts the power-transformed and standardized expression of all genes in the cluster, with the center lines denoting the clusters' means and standard deviations across developmental stages of *C. elegans* nematodes.

## Data Availability

The following previously published data sets were used: Dodd W, Tang L, Lone JC, Wimberly K, Wu CW, Consalvo C, Wright JE, Pujol N, Choe KP (2018) NCBI Gene Expression Omnibus ID GSE107704 (36). Role of SKN-1 in dpy-7 and osmotic gene induction. Finger F, Ottens F, Springhorn A, Drexel T, Proksch L, Metz S, Cochella L, Hoppe T (2019) NCBI Gene Expression Omnibus ID GSE124178 (37). RNA-seq: WT under proteotoxic stress conditions. Schreiner W, PPagliuso D, CGarrigues JM, Chen JS, Aalto AP, Pasquinelli AE (2019) NCBI Gene Expression Omnibus ID GSE132838 (38). RNA Sequencing of CTRL and Heat Stressed C. elegans [RNA-seq].

## Abbreviations

GUI: Graphical user interface
GO: Gene Ontology
PCA: Principal component analysis
RNA-Seq: RNA sequencing
MRN: Median ratio normalization
TMM: Trimmed mean of M-values
RLE: Relative log expression
WT: Wild type
KEGG: Kyoto Encyclopedia of Genes and Genomes

## References

[1] Martin M. Cutadapt removes adapter sequences from high-throughput sequencing reads. EMBnet.journal. 2011;17(1):10–2 Available from: https://journal.embnet.org/index.php/embnetjournal/article/view/200/479. Cited 4 Nov 2022.

[2] Bray NL, Pimentel H, Melsted P, Pachter L. Near-optimal probabilistic RNA-seq quantification. Nat Biotechnol 2016 345. 2016;34(5):525–7 Available from: https://www.nature.com/articles/nbt.3519. Cited 4 Nov 2022. 10.1038/nbt.3519 27043002

[3] Love MI, Huber W, Anders S. Moderated estimation of fold change and dispersion for RNA-seq data with DESeq2. Genome Biol. 2014;15(12):1–21 Available from: https://genomebiology.biomedcentral.com/articles/10.1186/s13059-014-0550-8. Cited 4 Nov 2022. 10.1186/s13059-014-0550-8 PMC4302049 25516281

[4] Soneson C, Love MI, Robinson MD. Differential analyses for RNA-seq: transcript-level estimates improve gene-level inferences. F1000Research. 2015;4:1521 /pmc/articles/PMC4712774/. 10.12688/f1000research.7563.1 PMC4712774 26925227

[5] Langmead B, Salzberg SL. Fast gapped-read alignment with Bowtie 2. Nat Methods 2012 94. 2012;9(4):357–9 Available from: https://www.nature.com/articles/nmeth.1923. Cited 2 Feb 2023. 10.1038/nmeth.1923 PMC3322381 22388286

[6] Liao Y, Smyth GK, Shi W. featureCounts: an efficient general purpose program for assigning sequence reads to genomic features. Bioinformatics. 2014;30(7):923–30 Available from: https://academic.oup.com/bioinformatics/article/30/7/923/232889. Cited 2 Feb 2023. 10.1093/bioinformatics/btt656 24227677

[7] Ritchie ME, Phipson B, Wu D, Hu Y, Law CW, Shi W, et al. limma powers differential expression analyses for RNA-sequencing and microarray studies. Nucleic Acids Res. 2015;43(7):e47–e47 Available from: https://academic.oup.com/nar/article/43/7/e47/2414268. Cited 5 Feb 2023. 10.1093/nar/gkv007 PMC4402510 25605792

[8] Law CW, Chen Y, Shi W, Smyth GK. Voom: Precision weights unlock linear model analysis tools for RNA-seq read counts. Genome Biol. 2014;15(2):1–17 Available from: https://genomebiology.biomedcentral.com/articles/10.1186/gb-2014-15-2-r29. Cited 5 Feb 2023 10.1186/gb-2014-15-2-r29 PMC4053721 24485249

[9] Bullard JH, Purdom E, Hansen KD, Dudoit S. Evaluation of statistical methods for normalization and differential expression in mRNA-Seq experiments. BMC Bioinformatics. 2010;11(1):1–13 Available from: https://bmcbioinformatics.biomedcentral.com/articles/10.1186/1471-2105-11-94. Cited 4 Nov 2022. 10.1186/1471-2105-11-94 PMC2838869 20167110

[10] Robinson MD, Oshlack A. A scaling normalization method for differential expression analysis of RNA-seq data. Genome Biol. 2010;11(3):1–9 Available from: https://genomebiology.biomedcentral.com/articles/10.1186/gb-2010-11-3-r25. Cited 4 Nov 2022. 10.1186/gb-2010-11-3-r25 PMC2864565 20196867

[11] Anders S, Huber W. Differential expression analysis for sequence count data. Genome Biol. 2010;11(10):1–12 Available from: https://genomebiology.biomedcentral.com/articles/10.1186/gb-2010-11-10-r106. Cited 4 Nov 2022. 10.1186/gb-2010-11-10-r106 PMC3218662 20979621

[12] Maza E, Frasse P, Senin P, Bouzayen M, Zouine M. Comparison of normalization methods for differential gene expression analysis in RNA-Seq experiments: A matter of relative size of studied transcriptomes. Commun Integr Biol. 2013;6(6):e25849 Available from: https://www.tandfonline.com/doi/abs/10.4161/cib.25849. Cited 4 Nov 2022. 10.4161/cib.25849 PMC3918003 26442135

[13] McInnes L, Healy J, Astels S. hdbscan: Hierarchical density based clustering. J Open Source Softw. 2017;2(11):205 Available from: http://joss.theoj.org/papers/10.21105/joss.00205. Cited 26 Jun 2020.

[14] Mimaroglu S, Yagci M. CLICOM: Cliques for combining multiple clusterings. Expert Syst Appl [Internet]. 2012;39(2):1889–901. [cited 2020 Jul 9]. Available from: https://www.sciencedirect.com/science/article/pii/S0957417411011705.

[15] Son YS, Baek J (2008). A modified correlation coefficient based similarity measure for clustering time-course gene expression data. Pattern Recognit Lett.

[16] Jaskowiak PA, Campello RJGB, Costa IG. On the selection of appropriate distances for gene expression data clustering. BMC Bioinformatics. 2014;15(S2):S2 Available from: https://bmcbioinformatics.biomedcentral.com/articles/10.1186/1471-2105-15-S2-S2. Cited 26 Jun 2020. 10.1186/1471-2105-15-S2-S2 PMC4072854 24564555

[17] Subramanian A, Tamayo P, Mootha VK, Mukherjee S, Ebert BL, Gillette MA, et al. Gene set enrichment analysis: A knowledge-based approach for interpreting genome-wide expression profiles. Proc Natl Acad Sci U S A. 2005;102(43):15545–50 Available from: https://www.pnas.org/doi/abs/10.1073/pnas.0506580102. Cited 4 Nov 2022. 10.1073/pnas.0506580102 PMC1239896 16199517

[18] Kanehisa M, Goto S. KEGG: kyoto encyclopedia of genes and genomes. Nucleic Acids Res. 2000;28(1):27–30.Available from: https://pubmed.ncbi.nlm.nih.gov/10592173/. Cited 4 Nov 2022. 10.1093/nar/28.1.27 PMC102409 10592173

[19] Ashburner M, Ball CA, Blake JA, Botstein D, Butler H, Cherry JM (2000). Gene Ontology: tool for the unification of biology. Nat Genet.

[20] Phipson B, Smyth GK. Permutation P-values should never be zero: Calculating exact P-values when permutations are randomly drawn. Stat Appl Genet Mol Biol. 2010;9(1):Article39 Available from: https://www.degruyter.com/document/doi/10.2202/1544-6115.1585/html. Cited 4 Nov 2022. 10.2202/1544-6115.1585 21044043

[21] Eden E, Lipson D, Yogev S, Yakhini Z. Discovering Motifs in Ranked Lists of DNA Sequences. PLOS Comput Biol. 2007;3(3):e39 Available from: https://journals.plos.org/ploscompbiol/article?id=10.1371/journal.pcbi.0030039. Cited 4 Nov 2022. 10.1371/journal.pcbi.0030039 PMC1829477 17381235

[22] Wagner F. The XL-mHG test for gene set enrichment. PeerJ Prepr [Internet]. 2017;5:e1962v3. [cited 2022 Nov 4]. Available from: https://peerj.com/preprints/1962.

[23] Kanehisa M, Furumichi M, Sato Y, Kawashima M, Ishiguro-Watanabe M. KEGG for taxonomy-based analysis of pathways and genomes. Nucleic Acids Res. 2022;51(D1):D587–92 Available from: https://academic.oup.com/nar/advance-article/doi/10.1093/nar/gkac963/6775388. Cited 4 Nov 2022. 10.1093/nar/gkac963 PMC9825424 36300620

[24] Carbon S, Douglass E, Good BM, Unni DR, Harris NL, Mungall CJ (2021). The Gene Ontology resource: enriching a GOld mine. Nucleic Acids Res.

[25] Virtanen P, Gommers R, Oliphant TE, Haberland M, Reddy T, Cournapeau D, et al. SciPy 1.0: fundamental algorithms for scientific computing in Python. Nat Methods. 2020;17(3):261–72 Available from: https://www.nature.com/articles/s41592-019-0686-2. Cited 4 Nov 2022. 10.1038/s41592-019-0686-2 PMC7056644 32015543

[26] Heyer LJ, Kruglyak S, Yooseph S. Exploring expression data identification and analysis of coexpressed genes. Genome Res. 1999;9(11):1106–15 Available from: http://genome.cshlp.org/content/9/11/1106.full. Cited 26 Jun 2020. 10.1101/gr.9.11.1106 PMC310826 10568750

[27] Harris CR, Millman KJ, van der Walt SJ, Gommers R, Virtanen P, Cournapeau D, et al. Array programming with NumPy. Nat 2020 5857825. 2020;585(7825):357–62. Available from: https://www.nature.com/articles/s41586-020-2649-2. Cited 4 Nov 2022. 10.1038/s41586-020-2649-2 PMC7759461 32939066

[28] Hunter JD (2007). Matplotlib: A 2D graphics environment. Comput Sci Eng.

[29] Lam SK, Pitrou A, Seibert S. Numba: A LLVM-based Python JIT Compiler. In: Proceedings of LLVM-HPC 2015: 2nd Workshop on the LLVM Compiler Infrastructure in HPC - Held in conjunction with SC 2015: The International Conference for High Performance Computing, Networking, Storage and Analysis [Internet]. Association for Computing Machinery; 2015. [cited 2022 Nov 4]. Available from: 10.1145/2833157.2833162.

[30] Mckinney W (2010). Data Structures for Statistical Computing in Python.

[31] Pedregosa F, Michel V, Grisel O, Blondel M, Prettenhofer P, et al. Scikit-learn: Machine Learning in Python. J Mach Learn Res. 2011;12(85):2825–30 Available from: http://jmlr.org/papers/v12/pedregosa11a.html. Cited 4 Nov 2022.

[32] Waskom ML. seaborn: statistical data visualization. J Open Source Softw. 2021;6(60):3021 Available from: https://joss.theoj.org/papers/10.21105/joss.03021. Cited 4 Nov 2022.

[33] Seabold S, Perktold J. statsmodels: Econometric and statistical modeling with Python. In: 9th Python in Science Conference [Internet]. 2010. [cited 2022 Nov 4]. Available from: http://statsmodels.sourceforge.net/.

[34] Lex A, Gehlenborg N, Strobelt H, Vuillemot R, Pfister H (2014). UpSet: Visualization of intersecting sets. IEEE Trans Vis Comput Graph.

[35] Davis P, Zarowiecki M, Arnaboldi V, Becerra A, Cain S, Chan J, et al. WormBase in 2022—data, processes, and tools for analyzing Caenorhabditis elegans. Genetics. 2022;220(4):iyac003 Available from: https://academic.oup.com/genetics/article/220/4/iyac003/6521733. Cited 13 Nov 2022. 10.1093/genetics/iyac003 PMC8982018 35134929

[36] Dodd W, Tang L, Lone JC, Wimberly K, Wu CW, Consalvo C, et al. A damage sensor associated with the cuticle coordinates three core environmental stress responses in caenorhabditis elegans. Genetics. 2018;208(4):1467–82 Available from: https://pubmed.ncbi.nlm.nih.gov/29487136/. Cited 30 Nov 2020. 10.1534/genetics.118.300827 PMC5887142 29487136

[37] Finger F, Ottens F, Springhorn A, Drexel T, Proksch L, Metz S, et al. Olfaction regulates organismal proteostasis and longevity via microRNA-dependent signalling Nature Metabolism. Nature Research. 2019;1:350–9 Available from: /pmc/articles/PMC6751085/?report=abstract. Cited 30 Nov 2020. 10.1038/s42255-019-0033-z PMC6751085 31535080

[38] Schreiner WP, Pagliuso DC, Garrigues JM, Chen JS, Aalto AP, Pasquinelli AE. Remodeling of the Caenorhabditis elegans non-coding RNA transcriptome by heat shock. Nucleic Acids Res. 2019;47(18):9829–41 Available from: https://pubmed.ncbi.nlm.nih.gov/31396626/. Cited 30 Nov 2020. 10.1093/nar/gkz693 PMC6765114 31396626

[39] Sloutsky R, Jimenez N, Swamidass SJ, Naegle KM. Accounting for noise when clustering biological data. Brief Bioinform [Internet]. 2013;14(4):423–36. [cited 2020 Jun 26]. Available from: https://academic.oup.com/bib/article-abstract/14/4/423/192812. 10.1093/bib/bbs057 23063929

[40] Houri-Zeevi L, Teichman G, Gingold H, Rechavi O. Stress resets ancestral heritable small RNA responses. eLife [Internet]. 2021;10. Available from: https://elifesciences.org/articles/65797. 10.7554/eLife.65797 PMC8021399 33729152

[41] Goecks J, Nekrutenko A, Taylor J, Afgan E, Ananda G, Baker D, et al. Galaxy: a comprehensive approach for supporting accessible, reproducible, and transparent computational research in the life sciences. Genome Biol. 2010;11(8):1–13 Available from: https://genomebiology.biomedcentral.com/articles/10.1186/gb-2010-11-8-r86. Cited 2 Feb 2023. 10.1186/gb-2010-11-8-r86 PMC2945788 20738864

[42] Afgan E, Baker D, Batut B, Van Den Beek M, Bouvier D, Ech M, et al. The Galaxy platform for accessible, reproducible and collaborative biomedical analyses: 2018 update. Nucleic Acids Res. 2018;46(W1):W537-44 Available from: https://academic.oup.com/nar/article/46/W1/W537/5001157. Cited 4 Nov 2022. 10.1093/nar/gky379 PMC6030816 29790989

[43] Spinozzi G, Tini V, Adorni A, Falini B, Martelli MP. ARPIR: automatic RNA-Seq pipelines with interactive report. BMC Bioinformatics. 2020;21(19):1–14 Available from: https://bmcbioinformatics.biomedcentral.com/articles/10.1186/s12859-020-03846-2. Cited 4 Nov 2022. 10.1186/s12859-020-03846-2 PMC7751108 33349239

[44] Srivastava H, Ferrell D, Popescu GV. NetSeekR: a network analysis pipeline for RNA-Seq time series data. BMC Bioinformatics. 2022;23(1):1–14 Available from: https://bmcbioinformatics.biomedcentral.com/articles/10.1186/s12859-021-04554-1. Cited 4 Nov 2022. 10.1186/s12859-021-04554-1 PMC8796424 35090393

[45] Marini F, Linke J, Binder H. ideal: an R/Bioconductor package for interactive differential expression analysis. BMC Bioinformatics. 2020;21(1):1–16 Available from: https://bmcbioinformatics.biomedcentral.com/articles/10.1186/s12859-020-03819-5. Cited 4 Nov 2022. 10.1186/s12859-020-03819-5 PMC7724894 33297942

[46] Zhu Q, Fisher SA, Dueck H, Middleton S, Khaladkar M, Kim J. PIVOT: Platform for interactive analysis and visualization of transcriptomics data. BMC Bioinformatics. 2018;19(6):1–8 Available from: https://bmcbioinformatics.biomedcentral.com/articles/10.1186/s12859-017-1994-0. Cited 4 Nov 2022. 10.1186/s12859-017-1994-0 PMC5756333 29304726

[47] Kucukural A, Yukselen O, Ozata DM, Moore MJ, Garber M. DEBrowser: Interactive differential expression analysis and visualization tool for count data 06 Biological Sciences 0604 Genetics 08 Information and Computing Sciences 0806 Information Systems. BMC Genomics. 2019;20(1):1–12 Available from: https://bmcgenomics.biomedcentral.com/articles/10.1186/s12864-018-5362-x. Cited 4 Nov 2022. 10.1186/s12864-018-5362-x PMC6321710 30611200

[48] Alexa ÃA, Rahnenführer J, Lengauer T. Improved scoring of functional groups from gene expression data by decorrelating GO graph structure. Bioinformatics. 2006;22(13):1600–7 Available from: https://academic.oup.com/bioinformatics/article-abstract/22/13/1600/193669. Cited 14 Jul 2020. 10.1093/bioinformatics/btl140 16606683

[49] Benjamini Y, Yekutieli D. The control of the false discovery rate in multiple testing under dependency. Ann Stat. 2001;29(4):1165–88. Available from: 10.1214/aos/1013699998https://projecteuclid.org/journals/annals-of-statistics/volume-29/issue-4/The-control-of-the-false-discovery-rate-in-multiple-testing/10.1214/aos/1013699998.full. Cited 29 Nov 2022.

[50] Yan M, Ye K. Determining the Number of Clusters Using the Weighted Gap Statistic. Biometrics [Internet]. 2007;63(4):1031–7. [cited 2020 Jun 26]. Available from: http://doi.wiley.com/10.1111/j.1541-0420.2007.00784.x. 10.1111/j.1541-0420.2007.00784.x 17425640

