# Supplementary figures and images for: RNAlysis: analyze your RNA sequencing data without writing a single line of code
Source: BMC Biol. 2023 Apr 7;21:74. doi: 10.1186/s12915-023-01574-6 (PMC10080885; doi:10.1186/s12915-023-01574-6)

Results of K-Medoids Clustering for n\_clusters=11, metric='yr1', power\_transform=True

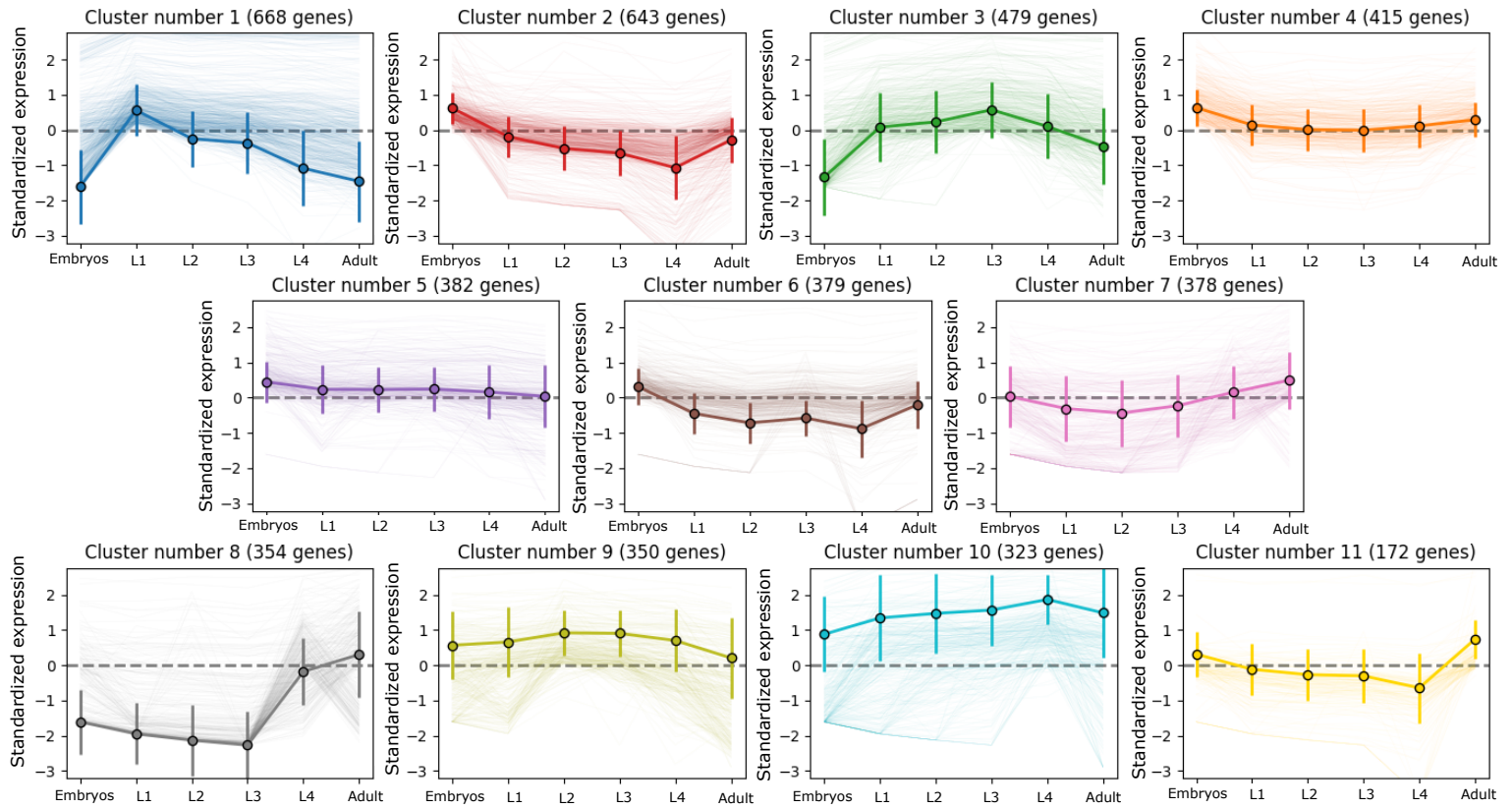

Supplement: Supplementary file 3 — Additional file 3: Figure S1. K-Medoids Clustering analysis of time-series gene expression data. Clustering analysis of the data using K-Medoids clustering, after selecting an appropriate number of clusters (K = 11) using the Gap Statistic method [50]. Clusters are sorted by their size. Each graph depicts the power-transformed and standardized expression of all genes in the cluster, with the center lines denoting the clusters' Medoids and standard deviations across developmental stages of C. elegans nematodes. [file 12915_2023_1574_MOESM3_ESM.pdf]
