# Supplementary material for: RNAlysis: analyze your RNA sequencing data without writing a single line of code
Source: BMC Biol. 2023 Apr 7;21:74. doi: 10.1186/s12915-023-01574-6 (PMC10080885; doi:10.1186/s12915-023-01574-6)

Results of HDBSCAN Clustering for min_cluster_size=75, min_samples = 1, metric='yr1', epsilon=0.0, method='eom' and power_transform=True

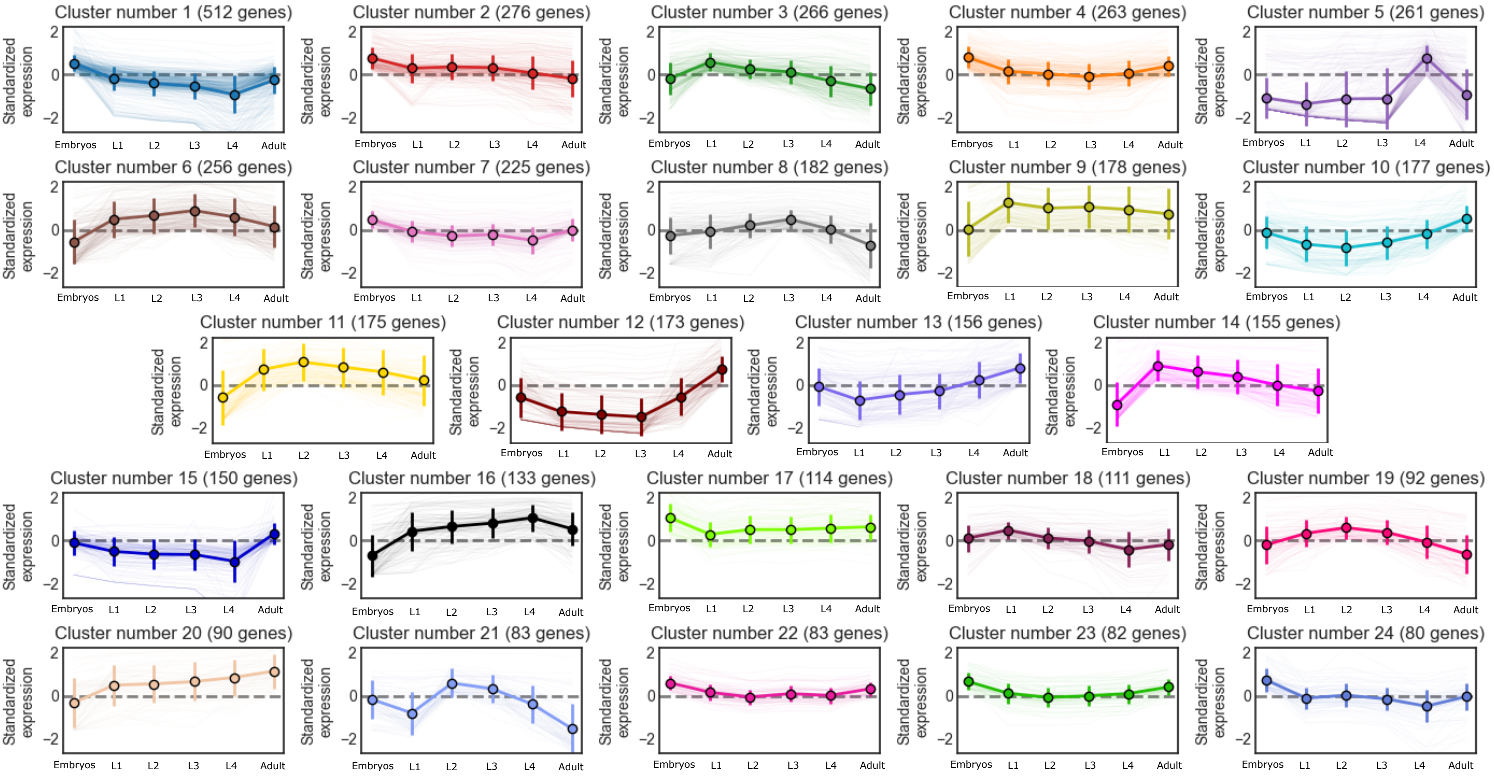

Supplement: Supplementary file 4 — Additional file 4: Figure S2. HDBSCAN Clustering analysis of time-series gene expression data. Clustering analysis of the data using HDBSCAN clustering, with a minimal cluster size of 75 [13]. Clusters are sorted by their size. Each graph depicts the power-transformed and standardized expression of all genes in the cluster, with the center lines denoting the clusters' means and standard deviations across developmental stages of C. elegans nematodes. [file 12915_2023_1574_MOESM4_ESM.pdf]
